# *Ready, Set, Change!* Development and usability testing of an online readiness for change decision support tool for healthcare organizations

**DOI:** 10.1186/s12911-016-0262-y

**Published:** 2016-02-24

**Authors:** Caitlyn Timmings, Sobia Khan, Julia E. Moore, Christine Marquez, Kasha Pyka, Sharon E. Straus

**Affiliations:** Li Ka Shing Knowledge Institute, St. Michael’s Hospital, 30 Bond Street, Toronto, M5B 1W8 Canada; University of Toronto, 563 Spadina Crescent, Toronto, M5S 2J7 Canada

**Keywords:** Readiness for change, Readiness assessment, Decision support tool, Tool development, Integrated knowledge translation, Usability testing, Implementation, Implementation support, Implementation planning

## Abstract

**Background:**

To address challenges related to selecting a valid, reliable, and appropriate readiness assessment measure in practice, we developed an online decision support tool to aid frontline implementers in healthcare settings in this process. The focus of this paper is to describe a multi-step, end-user driven approach to developing this tool for use during the planning stages of implementation.

**Methods:**

A multi-phase, end-user driven approach was used to develop and test the usability of a readiness decision support tool. First, readiness assessment measures that are valid, reliable, and appropriate for healthcare settings were identified from a systematic review. Second, a mapping exercise was performed to categorize individual items of included measures according to key readiness constructs from an existing framework. Third, a modified Delphi process was used to collect stakeholder ratings of the included measures on domains of feasibility, relevance, and likelihood to recommend. Fourth, two versions of a decision support tool prototype were developed and evaluated for usability.

**Results:**

Nine valid and reliable readiness assessment measures were included in the decision support tool. The mapping exercise revealed that of the nine measures, most measures (78 %) focused on assessing readiness for change at the organizational versus the individual level, and that four measures (44 %) represented all constructs of organizational readiness. During the modified Delphi process, stakeholders rated most measures as feasible and relevant for use in practice, and reported that they would be likely to recommend use of most measures. Using data from the mapping exercise and stakeholder panel, an algorithm was developed to link users to a measure based on characteristics of their organizational setting and their readiness for change assessment priorities. Usability testing yielded recommendations that were used to refine the *Ready, Set, Change! decision support tool* .

**Conclusions:**

*Ready, Set, Change! decision support tool* is an implementation support that is designed to facilitate the routine incorporation of a readiness assessment as an early step in implementation. Use of this tool in practice may offer time and resource-saving implications for implementation.

**Electronic supplementary material:**

The online version of this article (doi:10.1186/s12911-016-0262-y) contains supplementary material, which is available to authorized users.

## Background

To maximize the return on investments made in implementation initiatives and to ensure significant and sustainable impacts, healthcare organizations must rollout interventions that are known to be effective, using evidence-based and contextualized implementation processes [1–5]. Interventions refer to any coordinated set of activities designed to change targeted behavioural patterns, environments, or health outcomes [6, 7] and can include, but are not limited to, clinical practice guidelines, policies, health information technology, and evidence-based programs. Implementation is a complex process often resulting in unsuccessful attempts to adopt interventions. For instance, it has been estimated that $240 billion is invested per year in health and biomedical research globally; however, approximately 85 % of this funding is not optimally used as evidence is not adequately implemented in practice [8]. Furthermore, when initiatives are implemented, they often result in little to no meaningful practice change [9]. Contextual factors that surround a particular implementation effort can act to promote or hinder the implementation of evidence-based interventions [10].

Given the complexity of implementation, preparatory work to ehance implementation outcomes should be considered including establishing stakeholder buy-in [11], assessing barriers and facilitators to change [12], developing an implementation plan [11], and assessing and establishing organizational readiness for change [13–15]. Organizational readiness for change is defined as “the extent to which organizational members are both psychologically and behaviorally prepared to implement change” [16] and its assessment provides an opportunity to identify factors that may contribute to effective implementation. When readiness exists, an organization is more likely to accept the change, but when readiness is not established, the change is more likely to be rejected [17]. Furthermore, a readiness assessment affords an understanding of an organization’s level of readiness for change before resources are prematurely invested, and may help to avoid costly implementation errors [17].

**Fig. 1:**
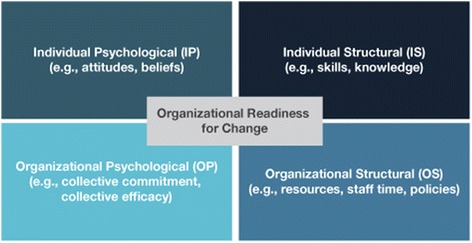
Organizational readiness for change constructs

Organizational readiness for change is composed of four underlying constructs (Fig. 1) that interact to determine an organization’s degree of readiness to implement a change intervention [16]:

1. Individual psychological (IP): Factors that reflect the extent to which individuals hold key beliefs regarding the potential change; recognize that a problem needs to be addressed; and agree with the changes required by individuals and the organization.
2. Individual structural (IS): Relevant dimensions related to the individual’s knowledge, skills, and ability to perform once the change is implemented.
3. Organizational psychological (OP): Relevant beliefs related to the organizational members’ collective commitment and collective efficacy.
4. Organizational structural (OS): Considerations related to human and material resources, communication channels, and formal policy.

Despite existing evidence on the importance of assessing readiness for change to promote successful implementation, many implementation teams do not assess, or do not accurately assess, readiness prior to implementation [18]. The underuse of readiness assessments is largely due to difficulty in selecting a valid, reliable, and appropriate readiness assessment measure [16, 18, 19]. The growing number of readiness assessment measures available for use makes it easier for implementers to access measures, provides variety, and may, in turn, increase the likelihood that implementers will use an existing measure instead of creating their own readiness assessment measure for one-time use; however, the large number of available measures also poses challenges. Challenges include difficulty in selecting a tool that is appropriate for a given setting and needs, that most measures have not been assessed for validity or reliability, and that many measures have been developed for specific settings so are not generalizable to other projects or contexts [19]. Given the number of measures available, selecting a measure that is appropriate for an organization’s particular needs and setting can be daunting and time-consuming for implementation teams. Additionally, it is unclear which, if any, of the underlying readiness for change constructs previously mentioned can be assessed by existing measures, rendering it difficult to accurately determine an organization’s level of readiness.

The task of selecting an appropriate instrument for assessing organizational readiness for change could be facilitated by the creation of a decision support tool for use during the early stages of implementation. To our knowledge, such a decision support tool does not exist for the readiness assessment phase. In this study, we aimed to develop and test the usability of a readiness assessment decision support tool using an end-user driven approach to promote the use of effective practices during the implementation planning phase.

## Methods

**Fig. 2:**
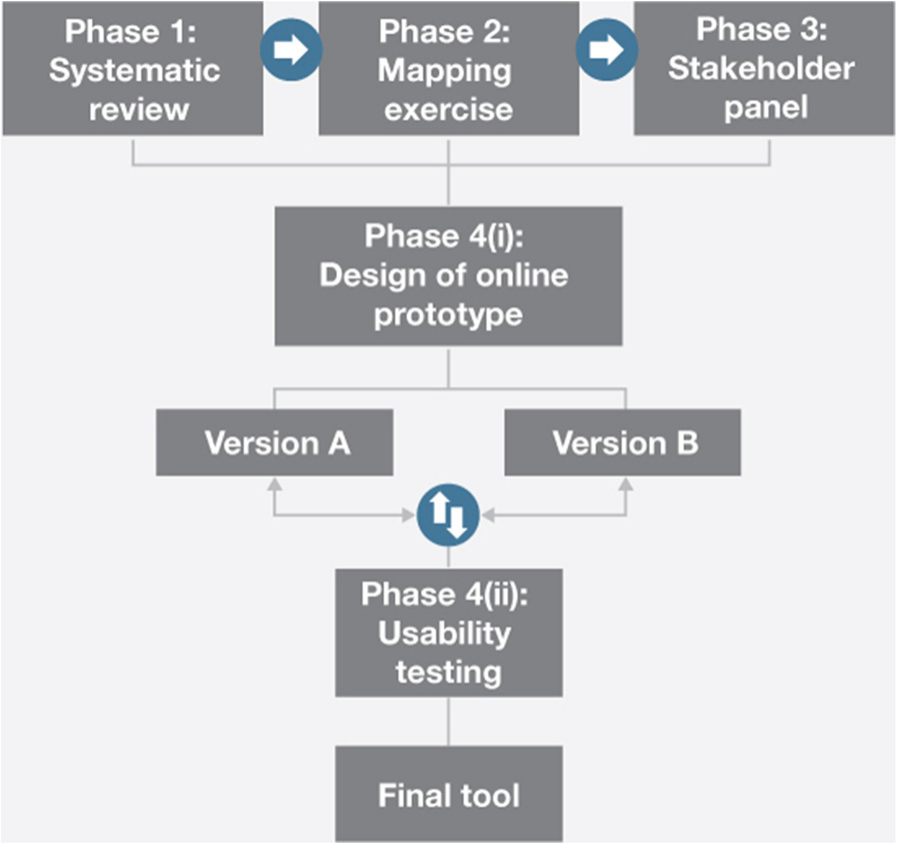
Tool development flow diagram

A multi-phase approach (Fig. 2) was used to develop the *Ready, Set, Change! decision support tool*. The full description of the methods was published previously [20] and only a brief description is provided here.

### Synthesizing available knowledge

#### Phase one: Selecting valid and reliable readiness assessment measures

Measures with demonstrated validity and reliability for assessing organizational readiness for change were identified from a recently completed systematic review of the theories and instruments used to assess organizational readiness for change in healthcare [21]. Of the 26 measures identified in the systematic review [21], we selected measures that were both valid *and* reliable (demonstrated through any measure of validity and reliability), and developed for use in healthcare settings (e.g., acute care, long-term care, public health). Measures designed to assess readiness for change in non-organizational settings (e.g., community) and measures that were not both valid *and* reliable were excluded.

### Phase two: Mapping items to a conceptual framework

Study investigators (including researchers and intermediaries supporting implementation activities) and research experts in organizational readiness for change were identified from existing professional networks using purposive sampling, and invited to participate in a mapping exercise to categorize the individual items of included readiness assessment measures according to key readiness constructs from an existing framework [16]. All items from the measures were mapped to one of the four readiness for change constructs independently by four reviewers [16]. Items that were inconsistent with any of the four constructs were categorized as ‘*other’*.

Reviewers conducted the mapping exercise independently; the intraclass correlation coefficient (ICC) was calculated to determine the degree of agreement among reviewers. An ICC assesses variability between quantitative measurements by accounting for both consistency of measures within raters and conformity of measures between raters [22]. It is a suitable statistic to measure the level of agreement among groups of raters when there is no “correct” response, and therefore only the absolute value of agreement is of interest [23]. Discrepancies were resolved through deliberations until consensus was reached [24]. The proportion of items measuring each of the four constructs of organizational change readiness (i.e., IP, IS, OP, or OS) and the ‘*other*’ category was calculated per readiness for change assessment measure using SPSS 22.0 software.

### Active engagement of end users in the tool development process

#### Phase three: Engaging a stakeholder panel

We engaged a stakeholder panel to complete a modified Delphi process [25]. The stakeholder panel consisted of individuals representing four categories of potential tool end users from various settings in the healthcare field (e.g., acute care, long-term care, public health, health policy) including: (1) implementers (e.g., clinicians, practitioners); (2) managers/administrators; (3) researchers; and (4) healthcare policymakers and funders. Stakeholders were recruited internationally via email using a purposive sampling approach to encourage equal representation of participants from each of the stakeholder groups. This was supplemented by snowball sampling until the desired number of participants was reached.

The modified Delphi process was conducted over two rounds. Participants were asked to rate the feasibility and relevance of included measures using a 7-point Likert scale (1 = strongly disagree; 7 = strongly agree). Participants were also asked to rate the likelihood they would recommend the use of the measure (e.g., to a colleague) using an 11-point scale (0 = not at all likely; 10 = extremely likely) for each of the included measures. A summary of results (Additional file 1) from the first round was distributed to participants by email, after which participants were asked to re-rank their responses. Stakeholder panel ratings for feasibility, relevance, and likelihood to recommend the use of the measure were analyzed using descriptive statistics [median, interquartile range (IQR)]. Stakeholders received a summary of the final results (Additional file 2).

### Phase four: Developing and testing the usability of an online decision support tool

Phase four was composed of two steps: (i) designing an online decision support tool prototype; and (ii) testing the usability of this prototype with potential end users.

**Fig. 3:**
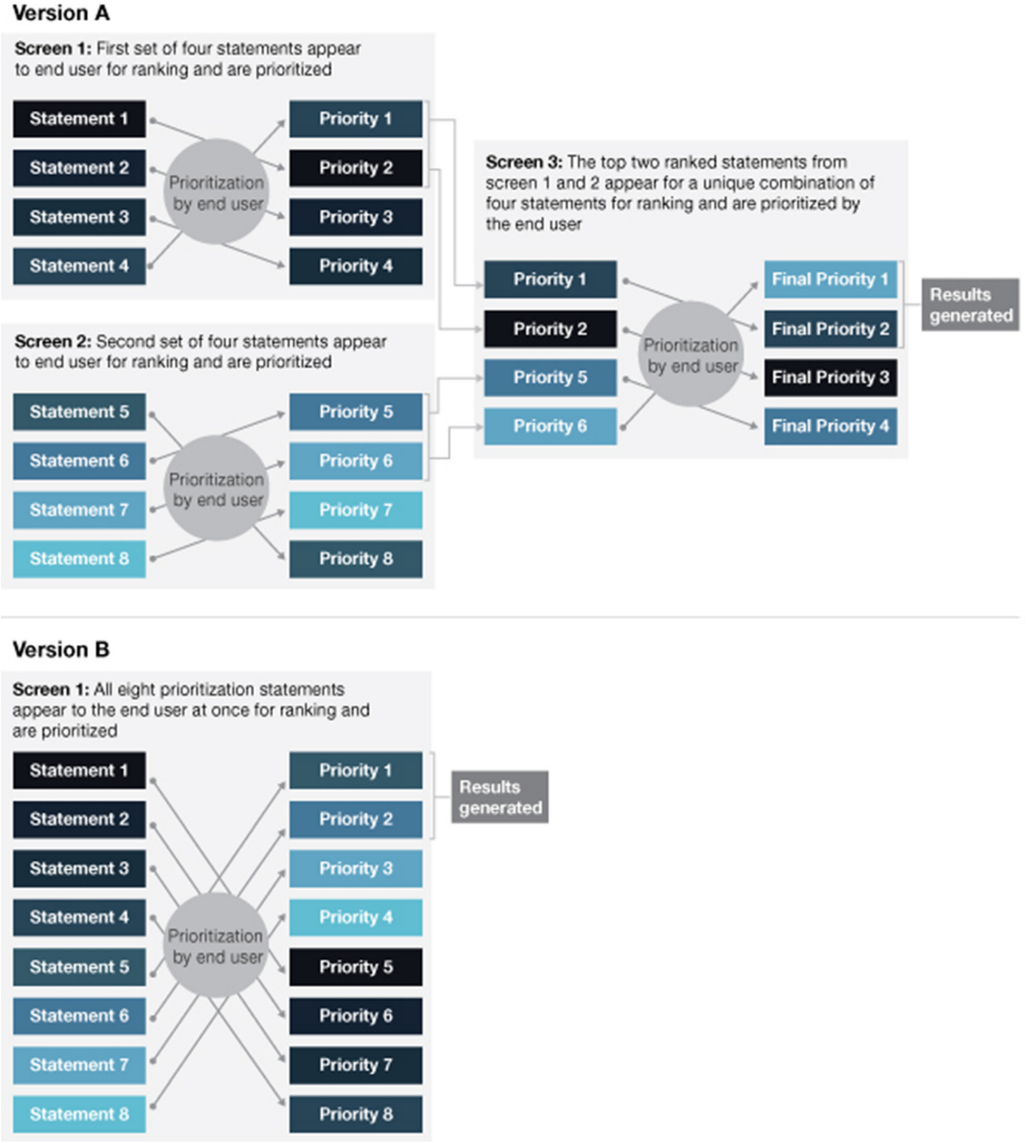
Schematic of decision support tool prototypes: Comparing approaches to the prioritization exercise in version **A** versus version **B**
(ii) Usability testing

(i) Designing the decision support tool prototype
  An algorithm was developed to link users’ organizational priorities (related to readiness for change assessment) with corresponding measures that contain items designed to evaluate these priorities. In developing the algorithm, we assumed that the ideal readiness assessment measure can be selected by ranking the importance of each of the four constructs of organizational readiness [16] to the organization, in order of most to least important. Recommended measures should include higher proportions of items addressing readiness constructs that align with organizational priorities [20]. Organizational priorities are represented by *prioritization statements -* a series of predetermined statements developed by the study team to typify each of the four underlying readiness constructs as defined by Holt et al’s framework [16]. The *prioritization statements* are ranked by the user in terms of importance in the context of their organizational setting. For example, *“it is important to assess how well staff in an organization work together to achieve a common goal”* is a statement designed to tap into priorities related to the readiness construct of OP. Each construct is represented by two *prioritization statements* for a total of eight statements.
  A series of screening questions (Additional file 3) was also developed to collect information on the end user’s implementation setting to determine the appropriateness of each measure for a given context. This information, together with user rankings of organizational readiness assessment priorities, is used to generate a list of potential measures that the end user could consider for use in their setting. The measures are presented along with median scores from phase three to provide end users with peer ratings of the recommended measure(s) on key domains (i.e., feasibility, relevance, and likelihood to recommend the measure).
  A preliminary version of the prototype was created using a staged ranking approach whereby the eight *prioritization statements* were presented to the user in groupings of four (version A). To test ease of use of the ranking approach, we developed an alternative prototype where all eight statements were presented to the user at once for ranking (version B) versus a staged ranking approach (version A). Fig. 3 provides a schematic of the prototype versions. The content of the *prioritization statements* was identical in each prototype version and was adapted from Holt et al’s definitions of the four readiness constructs (i.e., IP, IS, OP, OS) [16].

Both versions (A and B) of the decision support tool prototype were evaluated for usability [26, 27] with target end users [e.g., implementers (clinicians, practitioners); administrators/managers; researchers; and healthcare policymakers and funders]. We evaluated usability across two rounds. In the first round, we planned to test the usability of both versions (A and B) of the tool, and in a second round of testing, we planned to include only the version that was deemed to have fewer critical issues at the end of round one (as determined by the study team). Critical issues were defined as any issue observed during usability testing that directly hindered the user’s ability to interact with the tool. In round one of usability testing, participants were randomly assigned to use either version A or version B of the prototype; in round two, all participants were assigned the same tool version (the version with fewer critical issues detected).

Usability testing sessions were conducted during one-hour semi-structured interviews (Additional file 4) using a ‘think aloud’ methodology [28]. A ‘think aloud’ methodology involves the interviewer asking participants to verbalize their thoughts as they interact (e.g., rank the *prioritization statements*) with the tool or system being tested [28]. Participants were not provided with specific tasks/scenarios but rather were asked to approach the tool as if they were using it in their own organizational setting based on their implementation experience. All sessions were conducted online using WebEx live video conferencing software, and were audio recorded.

Audio recordings were transcribed verbatim, de-identified, and qualitatively analyzed by two analysts using a framework analysis approach [29]. Steps to framework analysis involve: familiarization of data; identification of a thematic framework based on a priori issues (i.e., usability measures and user experience) and emergent themes; application of the framework to data using textual codes (coding); and summarization of data according to catergories/themes (charting) [29].

### Ethics and consent

Ethical approval was obtained from St. Michael’s Hospital Research Ethics Board (REB #13-313). Informed consent was obtained from all participants.

## Results

### Phase one: Selecting valid and reliable readiness assessment measures

Nine valid and reliable readiness assessment measures were included in the readiness decision support tool (Table 1).

**Table 1:** List of measures included in *Ready, Set, Change! decision support tool*

#	Title of measure	Author	Year
M1^a^	Organizational Readiness for Change (Texas Christian University) [38]	Lehman et al.	2002
M2	Organizational Readiness to Change Assessment [39]	Helfrich et al	2009
M3	Long-Term Care (LTC) Readiness Tool [40]	Cherry et al	2011
M4	Team Climate Inventory [41]	Anderson & West	1994
M5	Measuring Practice Capacity for Change [42]	Bobiak et al	2009
M6	Perceived Organizational Readiness for Change [43]	Armenakis, Harris, & Mossholder	1993
M7	Organizational Change Questionnaire-Climate of Change, Processes, and Readiness [44]	Bouckenooghe et al	2009
M8	Organizational Information Technology Innovation Readiness Scale [45]	Snyder-Halpern	1996
M9	e-Health Readiness Measure [46]	Poissant & Curran	2007

^a^All included measures are survey instruments

### Phase two: Mapping items to a conceptual framework

There was excellent agreement [30] among the four independent reviewers who participated in the mapping exercise (ICC = 0.75, 95 % confidence interval [CI] [0.72, 0.78)]). Four of nine measures (44 %) included representation of all four constructs for assessing readiness. Items designed to assess factors related to the construct of “individual structural” (IS) were included in few of the nine measures (0 to 14 % of total items).

### Phase three: Engaging a stakeholder panel

Nineteen individuals participated in the stakeholder panel, with no attrition between rounds. Participant characteristics are provided in Table 2. Final scores of the stakeholder panel process related to feasibility, relevance, and likelihood to recommend are presented in Table 3 for each of the nine assessment measures identified in phase one.

**Table 2:** Demographics table for stakeholder panel (*N* = 19)

Target end user category	*n*
Healthcare policymakers and funders	7
Implementers (clinicians, practitioners)	4
Managers/administrators	4
Researchers	4
Country	*n*
Canada	15
United States	4

**Table 3:** Stakeholder panel ratings of feasibility, relevance, and likelihood to recommend for each included readiness to change measure [median, (IQR)]

Measure	Score [Median (IQR^a^)]		
	Feasibility^b^	Relevance^c^	Likelihood to recommend^d^
M1- Organizational Readiness for Change (Texas Christian University)	4.33 (1.67)	5.00 (1.00)	6.00 (2.75)
M2- Organizational Readiness to Change Assessment	5.17 (1.25)	5.00 (1.00)	6.00 (2.00)
M3- Long-Term Care (LTC) Readiness Tool	6.00 (0.33)	5.00 (1.00)	6.00 (2.00)
M4- Team Climate Inventory	6.00 (1.00)	5.00 (1.00)	7.00 (2.75)
M5- Measuring Practice Capacity for Change	4.00 (1.67)	3.00 (1.00)	3.00 (0.75)
M6- Perceived Organizational Readiness for Change	5.00 (1.33)	5.00 (1.00)	5.00 (2.75)
M7- Organizational Change Questionnaire-Climate of Change, Processes, and Readiness	6.00 (0.50)	6.00 (1.00)	8.00 (1.00)
M8- Organizational Information Technology Innovation Readiness Scale	5.00 (0.58)	5.00 (1.25)	5.00 (2.00)
M9- e-Health Readiness Measure	5.33 (0.67)	5.00 (0.25)	6.00 (0.75)

^a^ IQR = interquartile range (difference between 25th percentile and 75th percentile ratings)

^b^ Participants were asked to rate their level of agreement (*on a scale of 1 to 7 where 1 = strongly disagree and 7 = strongly agree*) with the following three statements related to feasibility: “*I think this measure can be used in a timely manner”*; “*I think this measure can be used without causing undue burden to existing resources (e.g., human resources, cost, etc.)”; and “overall, I understand how to use this readiness assessment measure”*

^c^ Participants were asked to rate their level of agreement (*on a scale of 1 to 7 where 1 = strongly disagree and 7 = strongly agree)* with the following statement related to relevance*: “I think this measure is relevant for assessing readiness for change”*

^d^ Participants were asked to rate the likelihood they would recommend the measure e.g., to a colleague or organization (*on a scale of 0 to 10 where 0 = not at all likely and 10 = extremely likely*) by responding to the following statement*: “What is the likelihood that you would recommend this measure?”*

Overall, the *Organizational Change Questionnaire-Climate of Change, Processes, and Readiness* measure (M7) was rated most highly by the stakeholder panel in all three categories of interest (median feasibility score = 6.00; median relevance score = 6.00; median likelihood to recommend score = 8.00). The *Measuring Practice Capacity for Change* measure (M5) received the lowest ratings of all measures reviewed (median feasibility score = 4.00; median relevance score = 3.00; median likelihood to recommend score = 3.00).

### Phase four: Developing and testing the usability of an online decision support tool

Fifteen usability testing sessions were conducted across two rounds, at which point it was determined that no further critical usability problems were uncovered. Characteristics of usability testing participants are provided in Table 4. Round one of usability testing included a total of 10 sessions (*n* = 5 sessions for version A and *n* = 5 sessions for version B) and round two included a total of five sessions conducted with version A. Specifically, since we observed fewer critical issues with version A, version A was selected for round two of testing and version B was discarded.

**Table 4:** Demographics table for usability testing participants (*N* = 15)

Target end user category	*n*
Implementers (clinicians, practitioners)	6
Managers/administrators	4
Researchers	4
Healthcare policymakers and funders	1
Country	*n*
Canada	13
Sweden	1
Switzerland	1

Four major themes were identified in the usability testing of the tools (across versions A and B): (1) perceived purpose of the tool; (2) content of the tool; (3) format of the tool; and (4) tool navigation.

#### Theme 1: Perceived purpose of the tool

The majority of participants (*n* = 13), demonstrated an understanding of the purpose of the *Ready, Set, Change! decision support tool* and identified advantages of its use. Many cited that the tool would aid in the decision-making process of selecting the most appropriate measure to assess readiness for a user’s organizational context and needs in a timely manner: “I really like this particular tool because it helps you think of that process and the impact – both organizationally on that system, and that individual level in terms of readiness and openness to change (*Participant 10, round 1*)”. A few participants (*n* = 4) expressed minor concerns about the tool (e.g., lack of direct access/availability of some of the recommended measures, and the appropriateness of some measures for their specific settings); however, the majority of participants indicated that they would recommend the use of the tool to others. One participant shared, “not only would I use it [the tool] but I could see myself, kind of, being a champion for the use of a tool like this in our organization” (*Participant 10, round 1*).

#### Theme 2: Content of the tool

Across both rounds of testing, the majority of participants indicated that the tool’s instructions were clear and easy to comprehend in both versions. Participants felt that the statements used to determine priorities were relevant to their organizations: “I would say that the choice of statements that I was asked to prioritize were very good statements that would need to be kept in mind when assessing readiness for change” (*Participant 14, round 2*). In round one, some participants reported some difficulty with comprehending content included in the tool (e.g., technical terminology such as “*change initiative*” or language used in some of the *prioritization statements*). Overall, most participants appreciated the user-centered features such as providing facts about the recommended measures such as the number of items included in the measure.

#### Theme 3: Format of the tool

Overall, participants felt that the layout of the tool followed a logical order: “it [the tool] took you through a logical set of steps to get to where you were at and you could actually see, by answering questions, you could see how the direction of where the tool is being chosen is going” (*Participant 8, round 1*). Participants further indicated that they liked how the information was presented (e.g., use of text bullets and graphs) and valued the use of colour-coded prompts as a means to distinguish the three sections of the tool: Section 1- Questions about the user’s organizational setting (blue); Section 2- Prioritization exercise (orange); and Section 3- Results (pink). Areas for improvement of the tool format were minor and were subsequently addressed, including: the use of a pie chart (versus a bar graph) to display results; improving visibility of the titles of the measure(s); and a preference for formatting the legends as open menus with the option “to close” (versus a default of hidden menus).

#### Theme 4: Tool navigation

Several participants commented that they appreciated having the tool navigation instructions provided at the beginning of the tool (both versions): “I think it’s valuable to have ‘how to navigate the tool’, it reduces frustration…” (P*articipant 6, round 1*). Others commented on the flexibility and ease of re-ordering prioritization statements (e.g., “drag and drop” options) such as, “I love the fact that you guys have the priority themes and then you just drag and drop – that’s a really great idea. It makes it really easy…” (*Participant 8, round 1*), and the inclusion of a progress bar as positive navigational features of the tool. A few participants commented on the benefits of the tool linking directly to the recommended measure(s) including one participant noting, “so I really like it that it actually leads you to the article [source of the measure]. That’s great” (*Participant 17, round 2*) or providing instructions on how to access the measure. Some participants expressed confusion with how to navigate back to the start of the tool after accessing their results during round one of usability testing. Following round one, this was addressed by the inclusion of additional instructions on how to exit versus restart the tool and was not identified as a critical issue in round two.

## Discussion

The current study used an end-user driven approach to develop a decision support tool for identifying valid, reliable, and appropriate organizational readiness for change assessment measures in practice. The *Ready, Set, Change! decision support tool* (http://readiness.knowledgetranslation.ca/) has been made freely available [31] to aid frontline implementers and decision-makers in selecting an appropriate readiness assessment measure for their needs.

To our knowledge, there are no decision support tools currently available to facilitate the process of selecting a valid, reliable, and appropriate readiness assessment measure. While training modules and guides are available to help implementation teams conduct a readiness assessment [32–34], we were unable to identify any decision support tools that facilitate selection of a readiness assessment tool for a particular setting. Acknowledging that healthcare organizations are increasingly being asked by funders or senior leadership to conduct a readiness assessment prior to implementation, and the time and fiscal constraints that many organizations continue to face, the process of selecting a valid and reliable readiness assessment measure must be streamlined to encourage its routine integration into practice [20]. Therefore, we believe our tool will contribute to the field of readiness for change assessment and complement existing efforts to aid implementers in understanding their organization’s degree of readiness for change by simplifying the measure selection process. Users may consider testing how application of the *Ready, Set, Change! decision support tool* has affected their implementation preparation process and outcomes and report on their evaluation.

The main strength of our study is that, through an integrated knowledge translation (KT) approach [35], we actively engaged end users at multiple stages of the tool development process. Involving potential end users in tool development is a critical step in ensuring the tool meets both functional goals (e.g., features, format, interface) and usability needs (e.g., end users’ requirements and information needs) [26]. The involvement of end users in our study has ensured that the product we created addresses the real-world needs of our target end users in selecting a valid, reliable, and appropriate readiness assessment measure in a timely manner. The tool development process also provided useful information about the composition of the individual readiness assessment measures. For example, we found that the ‘individual structural’ construct of organizational readiness for change is under-represented in available assessment measures. The literature indicates that it is important to include individual level constructs in an assessment of organizational readiness for change, as the extent to which an individual is inclined to accept or reject a plan to change the status quo affects overall organizational readiness for change [36]. Furthermore, although organizational members experience a shared context, individual perceptions of organizational readiness may vary [37]. New assessment measures in development, as well as existing measures, may consider including items that evaluate all four constructs that constitute organizational readiness for change to facilitate a more comprehensive evaluation of an organization’s degree of readiness.

There are some limitations to the approach used to develop and test the usability of the *Ready, Set, Change! decision support tool*. First, we used the results of a recently conducted systematic review that focused on measures applied in healthcare settings and excluded grey literature sources; thus, relevant readiness assessment measures could have been missed. Future iterations of the tool could utilize additional systematic reviews to identify readiness for change instruments developed for other settings and contexts to expand the spectrum of organizational readiness for change instruments available to end users. Second, individuals representing the ‘healthcare policymakers and funders’ end user category were under-represented in our sample for usability testing; however, these stakeholders are not typically involved directly with the implementation process. Convenience sampling was used to recruit participants to the various phases of this study, which may impact generalizability. Moreover, there may be limitations in the application of results to different cultural contexts; most of the included organizational readiness for change assessment measures were developed in the English language and for organizations in developed countries. We attempted to minimize these differences by recruiting only fluent English-speaking participants, specifically those who conduct their work primarily in the English language. In the future, we may add measures that pertain to different cultural contexts to expand the scope of the decision support tool beyond that of developed and English-speaking countries. Finally, at this stage of our study, we do not know the efficacy of the tool selection process offered by *Ready, Set, Change! decision support tool*. Future directions include testing these outcomes.

The development of the *Ready, Set, Change! decision support tool* has practical implications. As a decision support aid, *Ready, Set, Change!* may facilitate the use of readiness assessment measures in practice. We believe its use should be tested prospectively to determine impact on implementation. Additionally, a gap in the literature remains for how results of a readiness assessment should be interpreted and appropriate next steps for those organizations that are deemed not to be ready. Future studies may consider exploring this challenge.

## Conclusions

A decision support tool designed to guide implementers in healthcare settings in the selection of a valid, reliable, and appropriate readiness for change assessment measure was developed and tested for usability. The goal of *Ready, Set, Change! decision support tool* is to provide a rigorously developed implementation support to be used in practice during the planning stages of implementation. Next steps involve evaluating how use of the decision support tool affects implementation outcomes in a multi-site study involving hospitals in a Canadian province. The results of the prospective evaluation will provide information on tool utility and effectiveness, which can in turn, inform a strategy for how the tool can be refined and updated as additional readiness assessment measures that meet inclusion criteria are identified.

## Additional files

Additional file 1:
**Brief description: The summary of round one results shared with participants of the stakeholder panel.** (DOCX 140 kb) Additional file 2:
**Brief description: The summary of round two (final) results shared with participants of the stakeholder panel.** (DOCX 145 kb) Additional file 3:
**Brief description: The three screening questions users are asked in Section 1 (organizational setting and implementation context) of the online readiness for change decision support tool.** (DOCX 14 kb) Additional file 4:
**Brief description: The usability testing interview guide.** (DOCX 23 kb)

## Abbreviations

CI: confidence interval
ICC: intraclass correlation coefficient
IP: individual psychological
IQR: interquartile range
IS: individual structural
KT: knowledge translation
OP: organizational psychological
OS: organizational structural
