# Supplementary material for: Ready, Set, Change! Development and usability testing of an online readiness for change decision support tool for healthcare organizations
Source: BMC Med Inform Decis Mak. 2016 Feb 24;16:24. doi: 10.1186/s12911-016-0262-y (PMC4765048; doi:10.1186/s12911-016-0262-y)
Supplement: Additional file 1: — Brief description: The summary of round one results shared with participants of the stakeholder panel. (DOCX 140 kb) [file 12911_2016_262_MOESM1_ESM.docx]

**Readiness to Change: Stakeholder Panel**

**Round One Findings**

Presentation of the Readiness to Change Stakeholder Panel findings

This report presents the aggregate findings from the first round of the Stakeholder Panel. A total of 19 individuals participated in Round One. The response count and median value of the following response categories were computed for each included readiness to change measure: level of agreement with each of four statements; promoter score. The response count and median value of the accessibility score were computed for each of the four access scenarios presented in the rating tool. All aggregate findings are highlighted in the Summary of Findings section below.

What to do with these findings

Please review these findings to observe the general trend in responses and compare them to your own scores. The aggregate findings are meant to help you think about the justifications for your own ratings and why you may have rated similarly or differently from the majority of panel members. In Round Two, you will be given an opportunity to re-rate your responses and to justify why you kept your score the same or changed it in this round. Please keep in mind that when you respond, we would like you to think of the relevance of the 9 readiness assessment measures to the context of your own organization/work setting, organization types within your specific field of work, or organizations funded to implement best practices by your own organization. We encourage you to contact our team if you require information that might help you with your ratings in Round Two.

**Summary of Findings**

Feasibility

*Statement #1: Overall, I think this measure for assessing readiness for change can be used in a timely** manner.*

Median Response Count

|  | **Score*** | | | | | | |
| --- | --- | --- | --- | --- | --- | --- | --- |
| **Measure** | **1** | **2** | **3** | **4** | **5** | **6** | **7** |
| **1** | 1 | 3 | 3 | 2 | 2 | 7 | 1 |
| **2** | 0 | 3 | 1 | 3 | 6 | 3 | 2 |
| **3** | 0 | 0 | 0 | 0 | 5 | 1 | 3 |
| **4** | 0 | 0 | 1 | 4 | 5 | 4 | 5 |
| **5** | 0 | 2 | 3 | 3 | 2 | 5 | 4 |
| **6** | 0 | 2 | 0 | 3 | 3 | 5 | 6 |
| **7** | 0 | 0 | 0 | 2 | 4 | 8 | 5 |
| **8** | 0 | 2 | 0 | 2 | 7 | 4 | 3 |
| **9** | 0 | 0 | 0 | 2 | 8 | 4 | 3 |

*( 7 –point Likert Scale; 1 = strongly disagree; 7 = strongly agree)

**Note: We have defined timeliness in this regard as the amount of time taken to complete the measure, rather than occurring at a suitable or opportune time.

*Statement #2: Overall, I think this measure for assessing readiness for change can be used without causing undue burden to existing resources (e.g., human resources, cost, etc.).*

Median Response Count

|  | **Score*** | | | | | | |
| --- | --- | --- | --- | --- | --- | --- | --- |
| **Measure** | **1** | **2** | **3** | **4** | **5** | **6** | **7** |
| **1** | 0 | 3 | 2 | 5 | 4 | 3 | 2 |
| **2** | 0 | 2 | 2 | 3 | 5 | 3 | 3 |
| **3** | 0 | 0 | 0 | 0 | 3 | 1 | 5 |
| **4** | 0 | 1 | 1 | 2 | 5 | 5 | 5 |
| **5** | 0 | 1 | 1 | 3 | 5 | 3 | 6 |
| **6** | 0 | 1 | 0 | 3 | 2 | 7 | 6 |
| **7** | 0 | 0 | 1 | 2 | 4 | 6 | 6 |
| **8** | 0 | 1 | 2 | 2 | 7 | 2 | 4 |
| **9** | 0 | 0 | 1 | 3 | 5 | 3 | 5 |

*( 7 –point Likert Scale; 1 = strongly disagree; 7 = strongly agree)

*Statement #3: Overall, I understand how to use this readiness assessment measure.*

|  | **Score*** | | | | | | |
| --- | --- | --- | --- | --- | --- | --- | --- |
| **Measure** | **1** | **2** | **3** | **4** | **5** | **6** | **7** |
| **1** | 0 | 6 | 3 | 0 | 3 | 4 | 3 |
| **2** | 0 | 0 | 1 | 2 | 7 | 4 | 4 |
| **3** | 0 | 0 | 0 | 2 | 6 | 5 | 5 |
| **4** | 0 | 0 | 0 | 1 | 5 | 6 | 7 |
| **5** | 2 | 8 | 6 | 2 | 0 | 0 | 1 |
| **6** | 2 | 4 | 4 | 2 | 3 | 1 | 3 |
| **7** | 0 | 0 | 1 | 0 | 5 | 6 | 7 |
| **8** | 0 | 0 | 0 | 2 | 7 | 6 | 3 |
| **9** | 0 | 1 | 2 | 1 | 5 | 4 | 5 |

Median Response Count

*( 7 –point Likert Scale; 1 = strongly disagree; 7 = strongly agree)

Relevance

*Statement #4: Overall, I think this measure is relevant for assessing readiness for change in most health care organizational settings.*

Median Response Count

|  | **Score*** | | | | | | |
| --- | --- | --- | --- | --- | --- | --- | --- |
| **Measure** | **1** | **2** | **3** | **4** | **5** | **6** | **7** |
| **1** | 0 | 2 | 0 | 3 | 8 | 3 | 3 |
| **2** | 0 | 0 | 3 | 3 | 5 | 3 | 4 |
| **3** | 0 | 1 | 4 | 4 | 7 | 0 | 2 |
| **4** | 0 | 2 | 0 | 2 | 8 | 2 | 5 |
| **5** | 0 | 4 | 7 | 4 | 2 | 1 | 1 |
| **6** | 0 | 3 | 2 | 3 | 5 | 2 | 4 |
| **7** | 0 | 1 | 0 | 2 | 3 | 7 | 6 |
| **8** | 1 | 1 | 2 | 2 | 5 | 5 | 2 |
| **9** | 0 | 2 | 0 | 2 | 7 | 6 | 1 |

*( 7 –point Likert Scale; 1 = strongly disagree; 7 = strongly agree)

Promoter Score (i.e. likelihood to recommend)

*Likelihood you would recommend each measure (e.g., to a colleague or other organization interested in readiness assessment measures).*

Median Response Count

|  | **Score*** | | | | | | | | | | |
| --- | --- | --- | --- | --- | --- | --- | --- | --- | --- | --- | --- |
| **Measure** | **0** | **1** | **2** | **3** | **4** | **5** | **6** | **7** | **8** | **9** | **10** |
| **1** | 0 | 3 | 0 | 2 | 7 | 3 | 2 | 1 | 3 | 2 | 2 |
| **2** | 0 | 1 | 0 | 2 | 1 | 3 | 4 | 1 | 6 | 3 | 0 |
| **3** | 0 | 0 | 1 | 2 | 1 | 3 | 3 | 4 | 1 | 1 | 2 |
| **4** | 0 | 0 | 2 | 1 | 1 | 2 | 3 | 2 | 3 | 3 | 2 |
| **5** | 0 | 3 | 6 | 2 | 3 | 2 | 3 | 0 | 0 | 0 | 0 |
| **6** | 0 | 3 | 3 | 1 | 0 | 3 | 4 | 2 | 1 | 0 | 2 |
| **7** | 0 | 0 | 0 | 0 | 0 | 5 | 1 | 3 | 7 | 1 | 2 |
| **8** | 0 | 1 | 2 | 1 | 2 | 4 | 1 | 3 | 2 | 1 | 1 |
| **9** | 0 | 0 | 1 | 0 | 1 | 2 | 7 | 5 | 0 | 1 | 1 |

*(0 = not at all likely; 10 = extremely likely)

*(11- point Likert Scale; 0 = not at all likely; 10 = extremely likely)

Accessibility Score (i.e. likelihood to access measure)

*Likelihood that you would access a (hypothetical) measure based on each of the four scenarios below.*

Median Response Count

|  | **Score*** | | | | | | | | | | |
| --- | --- | --- | --- | --- | --- | --- | --- | --- | --- | --- | --- |
| **Scenario** | **0** | **1** | **2** | **3** | **4** | **5** | **6** | **7** | **8** | **9** | **10** |
| **If there was a cost associated with the measure (i.e., for purchase).** | 3 | 3 | 3 | 2 | 2 | 2 | 3 | 1 | 0 | 0 | 0 |
| **If the measure was embedded in an article (e.g., as an appendix, in a table).** | 0 | 0 | 1 | 0 | 0 | 5 | 2 | 1 | 5 | 1 | 4 |
| **If the measure was part of a larger tool kit (e.g., supported with examples of use, instructions for scoring).** | 0 | 0 | 0 | 1 | 0 | 0 | 1 | 1 | 6 | 2 | 8 |
| **If the measure was presented as a standalone document including instructions for use.** | 0 | 1 | 0 | 0 | 1 | 1 | 0 | 3 | 5 | 3 | 5 |

*(11- point Likert Scale; 0 = not at all likely; 10 = extremely likely)
