# Supplementary material for: Ready, Set, Change! Development and usability testing of an online readiness for change decision support tool for healthcare organizations
Source: BMC Med Inform Decis Mak. 2016 Feb 24;16:24. doi: 10.1186/s12911-016-0262-y (PMC4765048; doi:10.1186/s12911-016-0262-y)
Supplement: Additional file 2: — Brief description: The summary of round two (final) results shared with participants of the stakeholder panel. (DOCX 145 kb) [file 12911_2016_262_MOESM2_ESM.docx]

# Stakeholder Panel: Readiness for Change Assessment Measures

## Round Two (Final) Findings

### Organization of Stakeholder Panel findings

This report presents the aggregate findings from the second (final) round of the Stakeholder Panel on Readiness for Change Assessment Measures. All individuals who participated in Round One (N=19) also participated in Round Two (N= 19). Stakeholder responses are presented as response count and median for the level of agreement and promoter score given to statements related to feasibility, relevance, and ‘likelihood to recommend’ for each of the nine readiness for change assessment measures included in the exercise. The response count and median value are also presented for the ‘accessibility score’ computed for each of the four access scenarios presented in the exercise. All findings are organized in the graphs and tables presented in the Summary of Findings section below.

### How will these findings be used?

Round Two ratings are considered the final scores of the stakeholder panel process. These scores will be used to inform the development of the final readiness for change decision support tool prototype and will be shared with end users as part of the “results” section of the tool.

Many thanks for your participation as a member of the Stakeholder Panel. We greatly appreciate the time and effort you have devoted to this activity, and are certain that your contributions to our decision support tool will enhance the quality of our tool and the selection process for individuals and organizations interested in identifying valid, reliable, and appropriate readiness assessment measures for their settings.

## Summary of Findings

### Feasibility

***Statement #1: Overall, I think this measure for assessing readiness for change can be used in a timely* manner.***

Median Response Count

|  | **Score**** | | | | | | |
| --- | --- | --- | --- | --- | --- | --- | --- |
| **Measure** | **1** | **2** | **3** | **4** | **5** | **6** | **7** |
| **1** | 0 | 1 | 4 | 5 | 4 | 4 | 1 |
| **2** | 0 | 0 | 2 | 4 | 8 | 2 | 2 |
| **3** | 0 | 0 | 0 | 0 | 4 | 12 | 2 |
| **4** | 0 | 0 | 1 | 2 | 6 | 8 | 2 |
| **5** | 0 | 1 | 4 | 3 | 5 | 4 | 2 |
| **6** | 0 | 0 | 3 | 2 | 2 | 10 | 2 |
| **7** | 0 | 0 | 0 | 0 | 4 | 13 | 2 |
| **8** | 0 | 0 | 1 | 1 | 11 | 4 | 1 |
| **9** | 0 | 0 | 0 | 1 | 13 | 4 | 0 |

**( 7 –point Likert Scale; 1 = strongly disagree; 7 = strongly agree)

*Note: For this exercise, we have defined “timely” as the amount of time taken to complete the measure, rather than occurring at a suitable or opportune time.

***Statement #2: Overall, I think this measure for assessing readiness for change can be used without causing undue burden to existing resources (e.g., human resources, cost, etc.).***

Median Response Count

|  | **Score**** | | | | | | |
| --- | --- | --- | --- | --- | --- | --- | --- |
| **Measure** | **1** | **2** | **3** | **4** | **5** | **6** | **7** |
| **1** | 0 | 2 | 2 | 7 | 6 | 1 | 1 |
| **2** | 0 | 1 | 3 | 3 | 6 | 3 | 2 |
| **3** | 0 | 0 | 0 | 0 | 3 | 10 | 4 |
| **4** | 0 | 0 | 1 | 2 | 3 | 10 | 3 |
| **5** | 0 | 0 | 0 | 6 | 6 | 5 | 2 |
| **6** | 0 | 0 | 0 | 4 | 2 | 11 | 2 |
| **7** | 0 | 0 | 0 | 1 | 4 | 12 | 2 |
| **8** | 0 | 0 | 0 | 2 | 12 | 3 | 1 |
| **9** | 0 | 0 | 1 | 3 | 8 | 5 | 1 |

**( 7 –point Likert Scale; 1 = strongly disagree; 7 = strongly agree)

***Statement #3: Overall, I understand how to use this readiness assessment measure.***

Median Response Count

|  | **Score**** | | | | | | |
| --- | --- | --- | --- | --- | --- | --- | --- |
| **Measure** | **1** | **2** | **3** | **4** | **5** | **6** | **7** |
| **1** | 0 | 0 | 5 | 0 | 7 | 5 | 2 |
| **2** | 0 | 0 | 1 | 1 | 7 | 5 | 4 |
| **3** | 0 | 0 | 0 | 2 | 5 | 7 | 4 |
| **4** | 0 | 0 | 0 | 1 | 1 | 9 | 8 |
| **5** | 2 | 12 | 4 | 0 | 0 | 0 | 1 |
| **6** | 1 | 5 | 8 | 3 | 1 | 0 | 1 |
| **7** | 0 | 0 | 1 | 0 | 3 | 9 | 6 |
| **8** | 0 | 0 | 0 | 0 | 10 | 6 | 2 |
| **9** | 0 | 0 | 1 | 1 | 6 | 6 | 3 |

**(7 –point Likert Scale; 1 = strongly disagree; 7 = strongly agree)

### Relevance

***Statement #4: Overall, I think this measure is relevant for assessing readiness for change in most health care organizational settings.***

Median Response Count

|  | **Score**** | | | | | | |
| --- | --- | --- | --- | --- | --- | --- | --- |
| **Measure** | **1** | **2** | **3** | **4** | **5** | **6** | **7** |
| **1** | 0 | 0 | 0 | 1 | 8 | 6 | 2 |
| **2** | 0 | 0 | 2 | 1 | 6 | 3 | 3 |
| **3** | 0 | 0 | 3 | 3 | 7 | 1 | 1 |
| **4** | 0 | 2 | 0 | 1 | 6 | 6 | 2 |
| **5** | 0 | 3 | 8 | 3 | 2 | 1 | 0 |
| **6** | 0 | 1 | 1 | 6 | 5 | 3 | 1 |
| **7** | 0 | 0 | 0 | 2 | 3 | 8 | 4 |
| **8** | 0 | 0 | 2 | 3 | 7 | 2 | 2 |
| **9** | 0 | 0 | 0 | 2 | 10 | 4 | 0 |

**( 7 –point Likert Scale; 1 = strongly disagree; 7 = strongly agree)

### Promoter Score (i.e., likelihood to recommend)

***Statement: Likelihood you would recommend each measure (e.g., to a colleague or other organization interested in readiness assessment measures).***

Median Response Count

|  | **Score**** | | | | | | | | | | |
| --- | --- | --- | --- | --- | --- | --- | --- | --- | --- | --- | --- |
| **Measure** | **0** | **1** | **2** | **3** | **4** | **5** | **6** | **7** | **8** | **9** | **10** |
| **1** | 0 | 1 | 0 | 1 | 2 | 3 | 4 | 2 | 2 | 1 | 2 |
| **2** | 0 | 0 | 0 | 2 | 2 | 2 | 5 | 3 | 5 | 1 | 0 |
| **3** | 0 | 0 | 0 | 2 | 1 | 4 | 4 | 4 | 1 | 2 | 0 |
| **4** | 0 | 0 | 0 | 3 | 0 | 2 | 2 | 5 | 4 | 1 | 1 |
| **5** | 0 | 1 | 4 | 9 | 1 | 2 | 1 | 0 | 0 | 0 | 0 |
| **6** | 0 | 2 | 0 | 5 | 1 | 5 | 2 | 1 | 0 | 0 | 2 |
| **7** | 0 | 0 | 0 | 1 | 0 | 0 | 2 | 4 | 8 | 2 | 1 |
| **8** | 0 | 0 | 1 | 3 | 2 | 4 | 4 | 3 | 1 | 0 | 0 |
| **9** | 0 | 0 | 1 | 0 | 1 | 2 | 7 | 5 | 0 | 1 | 1 |

**(11- point Likert Scale; 0 = not at all likely; 10 = extremely likely)

### Accessibility Score (i.e., likelihood to access measure)

***Statement: Likelihood that you would access a (hypothetical) measure based on each of the four scenarios below.***

Median Response Count

|  | **Score**** | | | | | | | | | | |
| --- | --- | --- | --- | --- | --- | --- | --- | --- | --- | --- | --- |
| **Scenario** | **0** | **1** | **2** | **3** | **4** | **5** | **6** | **7** | **8** | **9** | **10** |
| **If there was a cost associated with the measure (i.e., for purchase).** | 1 | 1 | 4 | 4 | 3 | 2 | 1 | 1 | 0 | 0 | 0 |
| **If the measure was embedded in an article (e.g., as an appendix, in a table).** | 0 | 0 | 0 | 1 | 1 | 3 | 4 | 1 | 4 | 2 | 1 |
| **If the measure was part of a larger tool kit (e.g., supported with examples of use, instructions for scoring).** | 0 | 0 | 0 | 0 | 0 | 1 | 1 | 0 | 4 | 4 | 7 |
| **If the measure was presented as a standalone document including instructions for use.** | 0 | 1 | 0 | 0 | 0 | 0 | 1 | 1 | 6 | 6 | 2 |

**(11- point Likert Scale; 0 = not at all likely; 10 = extremely likely)
