# Supplementary material for: Ready, Set, Change! Development and usability testing of an online readiness for change decision support tool for healthcare organizations
Source: BMC Med Inform Decis Mak. 2016 Feb 24;16:24. doi: 10.1186/s12911-016-0262-y (PMC4765048; doi:10.1186/s12911-016-0262-y)
Supplement: Additional file 3: — Brief description: The three screening questions users are asked in Section 1 (organizational setting and implementation context) of the online readiness for change decision support tool. (DOCX 14 kb) [file 12911_2016_262_MOESM3_ESM.docx]

Section 1: Organizational setting and implementation context

Question 1: Which of the following *best* describes your organizational setting (i.e., the setting for change implementation)?

- Health care
- Public health and social services
- Education
- Government or government agency
- Research/academia
- Non-governmental/ Non-profit organization
- Industry
- Other

Question 2: To your knowledge, is your intervention of interest an eHealth intervention (e.g., electronic health record system, telemedicine, virtual health care team, consumer health informatics, etc.)?

- Yes
- No

Question 3: To your knowledge, has a plan already been developed for *how* your intervention of interest will be implemented in your organization? This question applies to both eHealth and non-eHealth interventions.

- Yes
- No
