# Supplementary material for: Ready, Set, Change! Development and usability testing of an online readiness for change decision support tool for healthcare organizations
Source: BMC Med Inform Decis Mak. 2016 Feb 24;16:24. doi: 10.1186/s12911-016-0262-y (PMC4765048; doi:10.1186/s12911-016-0262-y)
Supplement: Additional file 4: — Brief description: The usability testing interview guide. (DOCX 23 kb) [file 12911_2016_262_MOESM4_ESM.docx]

| **Readiness Decision Support Tool**  *Participant will be given the login information to access WebEx Meeting by email prior to starting usability testing.*  Please take a few minutes to look over and review the tool. Please “think aloud” as you are doing so and provide feedback (i.e., to say what you are looking at, what you are trying to do, and what you are thinking).  **Provide time for participant to review tool and provide feedback.** |
| --- |

| **Follow-up Questions (themes & questions)** | |
| --- | --- |
| **Purpose of Tool** | |
| **1.** | In 1-2 sentences, how would you describe the purpose of this decision support tool? |
| **2.** | Do you feel this tool would be useful/beneficial to you or your organization? Why/why not?  **Prompt:** Can you describe some of the advantages of using such a tool?  Can you describe some of the disadvantages of using such a tool? |
| **3.** | Would this tool help you make a decision regarding the selection of measures to assess readiness for change?  **Prompt:** Are there any specific aspects of the tool that may contribute to/hinder your decision? |
| **4.** | Do you think the output you get from the tool is worth the time it takes to complete the tool? |
| **5.** | Would you be inclined to use a tool like this in your organization setting? Why/why not? |
| **6.** | Would you recommend the use of this tool to others? Why/why not? |
| **Content** | |
| **7.** | Did you find the questions to be clear and easy to understand? Why/why not?  **Prompt:** Was it easy/difficult to know what each question was asking of you? Why/why not?  **Prompt:** In your opinion, is the language too complicated? |
| **8.** | Did the statements make sense/feel applicable to your setting and/or how you identify priorities related to change implementation? |
| **9.** | Is there anything about the tool in terms of content that you would change (e.g., reword, add or remove)?  **Prompt**: If so, what? Why? |
| **10.** | Is there any information missing from the tool that you would like to see? |
| **Format& Navigation** | |
| **11.** | Does the flow of the tool make sense?  **Prompt:** Does the order of the questions follow a logical progression?  **Prompt:** If not, how would you like the information organized or presented? |
| **12.** | Were the results (measures) displayed in a way that was useful and made sense?  **Prompt:** If not, how would you like the results to be displayed or presented?  **Prompt:** Would it be beneficial for the tool to link directly to the measures? |
| **13.** | Was the tool laid out in a way that made sense to you? Why/why not? |
| **14.** | Did you find the tool visually appealing (e.g., spacing and font)? Why/why not? |
| **Other** | |
| **15.** | Do you have any other feedback, comments or concerns about the decision support tool that you would like to share? |
| **Wrap Up** | |
| Turn off audio recorder.  Thank participant for their time.  Collect contact details for remuneration purposes (i.e., full name, address, email address, phone number). | |
